# Optimizing whole-body MRI for early cancer detection in Li-Fraumeni syndrome: a prospective bicentric study

**DOI:** 10.1007/s00330-025-11880-y

**Published:** 2025-10-21

**Authors:** Myriam Margareta Keymling, Felix Tobias Kurz, Tristan Anselm Kuder, Sebastian Bickelhaupt, Thomas Hielscher, Robert Hog, Theresa Mokry, Tawfik Moher Alsady, Sarah Schott, Christian Kratz, Diane Miriam Renz, Heinz-Peter Schlemmer

**Affiliations:** 1 Division of Radiology, German Cancer Research Center (DKFZ), Heidelberg, Germany; 2 Division of Neuroradiology, Geneva University Hospitals, Geneva, Switzerland; 3 Department of Medical Physics in Radiology, German Cancer Research Center (DKFZ), Heidelberg, Germany; 4 Institute of Radiology, University Hospital Erlangen, Erlangen, Germany; 5 Division of Biostatistics, German Cancer Research Center (DKFZ), Heidelberg, Germany; 6 Department of Diagnostic and Interventional Radiology, Heidelberg University Hospital, Heidelberg, Germany; 7 Department of Neuroradiology, University Hospital of Giessen and Marburg, Campus Marburg, Marburg, Germany; 8 Department of Obstetrics and Gynecology, Heidelberg University Hospital, Heidelberg, Germany; 9 Department of Pediatric Hematology and Oncology, Hannover Medical School, Hanover, Germany; 10 Department of Pediatric Radiology, Institute of Diagnostic and Interventional Radiology, Hannover Medical School, Hanover, Germany

**Keywords:** Whole-body, Li-Fraumeni syndrome, Magnetic resonance imaging, Protocol optimization, Surveillance

## Abstract

**Objectives:**

Annual whole-body MRI (WB-MRI) is recommended for early cancer detection in individuals with Li-Fraumeni syndrome (LFS). However, there is no agreement on a standardized MRI protocol. This study evaluated the diagnostic performance of different MRI sequences to suggest an optimized protocol for LFS surveillance.

**Materials and methods:**

In this prospective bicentric study, 113 participants with LFS underwent annual WB-MRI and were included in the analysis. The protocol comprised turbo-spin echo (TSE) T1-weighted and inversion-recovery T2-weighted (TIRM) images of the whole body in coronal orientation, and T2-weighted (HASTE), diffusion-weighted (DWI), and T1-weighted DIXON images (pre- and post-contrast agent administration) from head to thighs in axial orientation. An additional fluid-attenuated inversion recovery (FLAIR) sequence imaged the skull only. Initial clinical interpretation was conducted by staff radiologists. The visibility of reported mass lesions was independently graded in all sequences by three experienced radiologists using a Likert scale. Sequence combinations were compared to inform the design of an optimal MRI protocol.

**Results:**

Over 30 months, 189 WB-MRI examinations were performed in 113 participants (mean age 40 years, ±12.7 years [standard deviation], 91 women). 188 mass lesions were detected and confirmed as malignant (*n* = 38), benign (*n* = 120) or ambiguous (*n* = 30). In the multi-reader analysis, all new malignant lesions could have been detected by a combination of cranial FLAIR, whole-body DWI, and whole-body HASTE in the axial direction.

**Conclusion:**

A shortened, contrast-agent-free WB-MRI protocol combining cranial FLAIR, WB-HASTE, and WB-DWI promises to be an effective and patient-friendly approach for annual cancer surveillance in LFS.

**Key Points:**

***Question*** *Annual whole-body MRI (WB-MRI) is recommended for early cancer detection for individuals with Li-Fraumeni syndrome (LFS), but a standardized sequence protocol has yet to be established*.

***Findings*** *The combination of cranial FLAIR, whole-body HASTE, and whole-body DWI in the axial plane enabled visualization of all newly developed malignant lesions in our study cohort*.

***Clinical relevance*** *A shortened, standardized WB-MRI protocol enables efficient, sensitive early cancer detection in individuals with LFS, minimizing patient burden by reducing examination time and contrast agent use. This approach may improve surveillance participation while enhancing comparability across centers*.

**Graphical Abstract:**

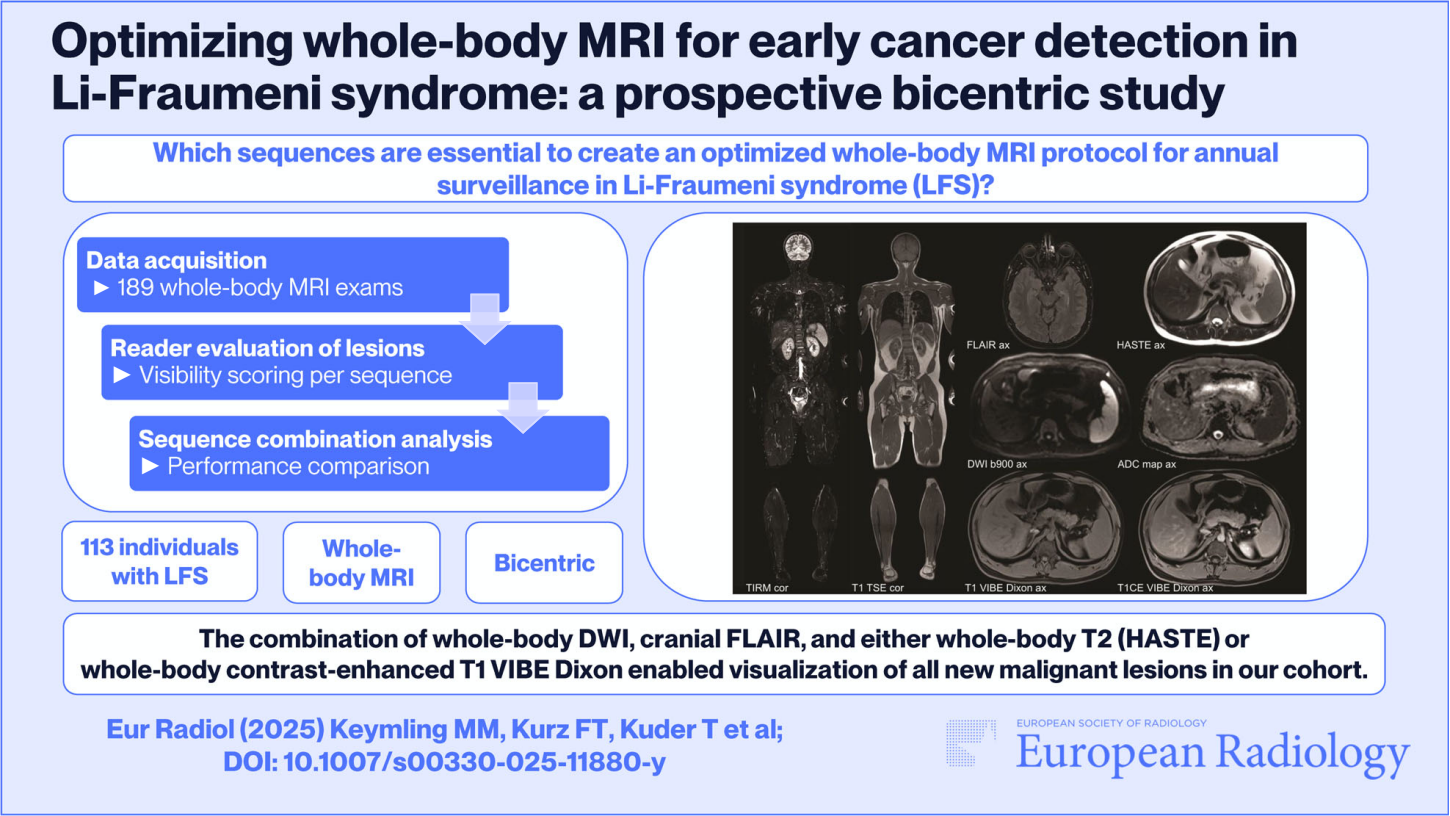

## Introduction

Li-Fraumeni syndrome (LFS) is a rare cancer predisposition syndrome caused by pathogenic variants in the *TP53* gene. Affected individuals face an exceptionally high risk of developing cancer, with a lifetime probability of ~50% by age 40 and up to 90% by age 60 [1]. The core tumor spectrum includes early-onset breast cancer, soft tissue and bone sarcomas, adrenocortical carcinomas, and brain tumors. In addition, a wide range of various other tumor types have been associated with LFS [2, 3].

Extensive surveillance strategies for LFS have been proposed for detecting tumors at early, treatable stages [4–6], most of which are based on the Toronto protocol developed in 2001 [7]. The latest version from 2017 includes regular physical, dermatological, and gastroenterological exams, along with three annual MRI examinations of the whole body, head, and breast [7, 8].

Among these, whole-body MRI is considered a cornerstone due to its ability to cover the entire body within a single session while avoiding the risks associated with radiation exposure [9].

After various studies had shown the suitability of WB-MRI for cancer detection from childhood onward [10–12] and indicated its utility in LFS [7, 13, 14], employed MRI protocols remained highly variable. An important step toward harmonization was the 2020 international ONCO-RADS consensus recommendation, which proposed a standardized WB-MRI protocol for LFS to be clinically validated [15]. A systematic evaluation of sequences could substantiate these guidelines and identify essential sequences to potentially reduce examination duration.

A particular concern in lifelong WB-MRI surveillance for individuals with LFS is the regular administration of gadolinium-based contrast agents, given their potential side effects and impact on patient adherence to surveillance [16].

Diffusion-weighted imaging (DWI), a contrast-agent-free technique sensitive to the densely packed cells of tumors [17], has been used for cancer detection within WB-MRI protocols before [18, 19]. Although some studies have even already integrated DWI in LFS surveillance [14, 15], its specific diagnostic performance compared to other sequences remains unclear.

This study aimed to address these gaps by evaluating a WB-MRI protocol in a German LFS cohort, with a focus on the performance of individual sequences. We hypothesized that a standardized, abbreviated protocol incorporating only contrast-free sequences, particularly DWI, would maintain a high sensitivity while potentially reducing examination burden and improving clinical feasibility.

## Materials and methods

### Study design and setting

**Fig. 1:**
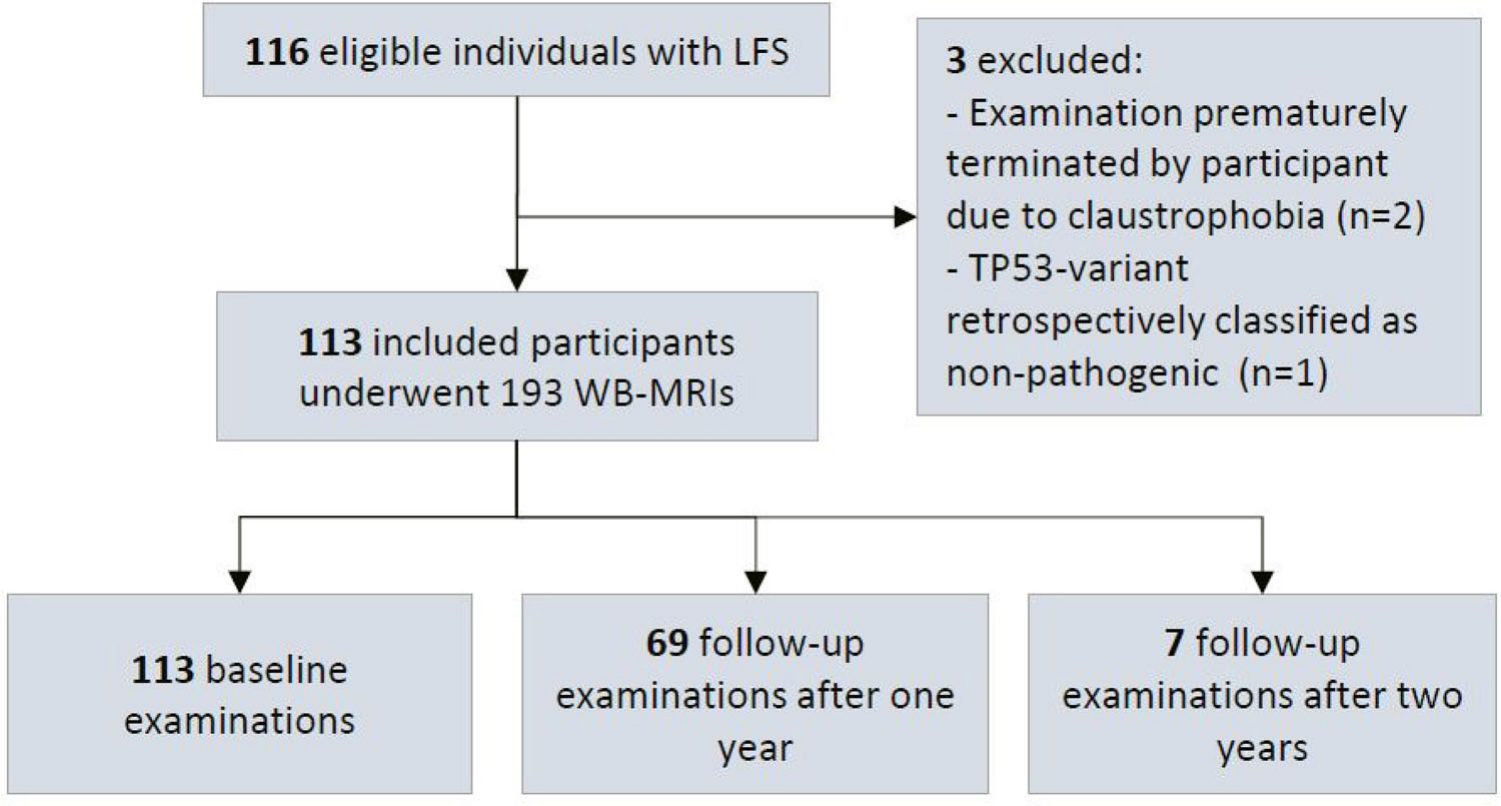
Flowchart with an overview of the study cohort

This ongoing prospective bicentric study was approved by the local institutional review boards in both study sites at the German Cancer Research Center in Heidelberg (S-131/2020) and at Hannover Medical School in Hanover (9196_B0_K_2020). It was conducted in accordance with the Declaration of Helsinki. Written informed consent was obtained from all participants prior to inclusion. As a subproject of the ADDRess (“Abnormal DNA Damage Response”) consortium, the study received financial support for personnel expenditure by the German Federal Ministry of Education and Research (BMBF) (01GM1909E). The BMBF had no influence on the study design, collection, and analysis of data. Participation was possible for all individuals with a confirmed pathogenic *TP53* mutation. Exclusion criteria included severe claustrophobia or MR unsafe implants. Participants were offered annual WB-MRI at either study site. This analysis includes 113 individuals examined between July 01, 2020, and December 31, 2022 (Fig. 1).

### WB-MRI protocol

WB-MRI examinations were performed at two 1.5-Tesla scanners (“Magnetom Aera” and “Magnetom Avanto”; Siemens) using a head/neck coil, two body array coils, a peripheral angio coil, and the spine coil embedded in the scanner bed. The protocol, adapted from previously published publications on LFS surveillance [14, 15], was developed in collaboration of scientists from both sites. It was tested and optimized extensively in both study sites prior to the beginning of the study. Thorough staff training ensured uniform examination quality across sites.

**Fig. 2:**
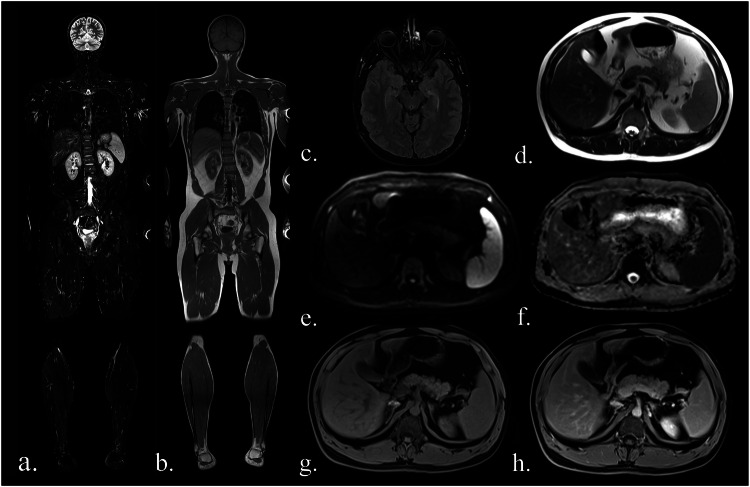
Sequences included in the study MRI protocol: (**a**) coronal TIRM (turbo inversion recovery magnitude) sequence, (**b**) coronal T1-weighted sequence, (**c**) axial FLAIR (fluid-attenuated inversion recovery) sequence (head only), (**d**) axial T2-weighted fast spin-echo sequence (HASTE; Siemens), (**e**) axial diffusion-weighted imaging (b-value 900), (**f**) an axial ADC map, (**g**) axial T1-Dixon water-only images before contrast agent and (**h**) after administration of contrast agent. Images were cropped to allow all sequences to be displayed

The protocol encompassed a broad range of MRI sequences with a total acquisition time of 77.5 min, including a contrast-enhanced T1 VIBE Dixon sequence using Gadoteridol (ProHance®; Bracco). To streamline acquisition, sequence order and acquisition blocks were adjusted to require only one B0 shim for DWI and T1 VIBE Dixon. DWI comprised 6 stations with 35 slices each, totaling an acquisition time of 24 min. As the standard single-shot DWI sequence tends to produce artifacts in the neck region, an additional DWI with slice-specific dynamic shimming was used in this area. For an overview of all sequences and further technical details, see Table 1; for details on DWI, Table 2. An exemplary WB-MRI examination is depicted in Fig. 2. Since images were cropped, we provided examples of uncropped images in Supplementary Fig. 1.

**Table 1:** Technical details of the MRI study protocol

Sequence name	Area of coverage	Orientation	TE (ms)	TR (ms)	FOV (mm^2^)	Matrix size	In-plane resolution (mm^2^)	Interpolation	Slice thickness (mm)
DWI (b-value 900 s/mm^2^)	Head to thighs	Axial, MPR → coronal	56	7500	500 × 342	164 × 164	1.5 × 1.5	On	6
DWI ishim	Neck	Axial	64	5250	460 × 288	128 × 128	1.8 × 1.8	On	6
T1 VIBE Dixon pre-contrast	Head to thighs	Axial	2.39/4.77	6.69	500 × 360	320 × 240	1.6 × 1.6	Off	3
T1 TSE	Whole body	Coronal	8.4	690	500 × 375	384 × 288	1.3 × 1.3	Off	4
T2 HASTE	Head to thighs	Axial	80	1200	500 × 344	320 × 256	0.8 × 0.8	On	6
TIRM	Whole body	Coronal	56	4590	500 × 375	320 × 240	0.8 × 0.8	On	4
FLAIR	Head	Axial	120	7290	250 × 195	256 × 218	0.5 × 0.5	On	4
T1 VIBE Dixon post-contrast	Whole body	Axial	2.39/4.77	6.69	500 × 360	320 × 240	1.6 × 1.6	Off	3

Sequence name	Slice gap (mm)	Bandwidth (Hz/Px)	Acquisition time (min)	Number of stations	Number of slices	Phase-encoding direction	Flip angle	IPAT	Ref. Lines	Respiratory control
DWI (b-value 900)	0	2540	24	6	35	A » P	90–180°	2	42	Free breathing
DWI ishim	0	1776	3.5	1	35	A » P	90–180°	2	32	Free breathing
T1 VIBE Dixon pre-contrast	0	470	3	6	72	A » P	10°	4	24	Station 2–4: Breath-hold; Station 1 and 5–6: Free breathing
T1 TSE	0.4	241	17	7	65	F » H	Refocusing 153–160°	2	30	Free breathing
T2 HASTE	0	539	9	6	35	A » P	180°	2	30	Breath-hold
TIRM	0.4	233	12.5	7	65	F » H	Refocusing 136–140°	3	27	Free breathing
FLAIR	0.4	131	3.5	1	40	R » L	Refocusing 150°	2	54	Free breathing
T1 VIBE Dixon post-contrast	0	470	5	10–11, depending on patient size	72	A » P	10°	4	24	Station 2–4: Breath hold; Station 1 and 5–10/11: Free breathing

T1 VIBE Dixon, if not otherwise specified, refers to four acquired sets of images: in-phase, opposed-phase, water-only, and fat-only images. In-plane resolution refers to the final image resolution, including interpolation, if turned on

*TE* echo time, *TR* repetition time, *FOV* field of view, *DWI* diffusion-weighted imaging, *MPR* multiplanar reformation, *VIBE* volumetric interpolated breath-hold, *TSE* turbo spin-echo, *HASTE* half Fourier-acquisition single-shot turbo spin echo, *TIRM* turbo inversion recovery magnitude, *FLAIR* fluid-attenuated inversion recovery, *IPAT* integrated parallel acquisition techniques, *A* anterior, *P* posterior, *F* foot, *H* head, *Ref. Lines* reference lines

**Table 2:** Additional parameters for DWI

Fat suppression	SPAIR
Phase partial Fourier	Off
Acceleration mode	GRAPPA
Acceleration factor phase-encoding	2
Diffusion mode	3D Diagonal
Diffusion scheme	Monopolar
b-values	0, 50, 900 s/mm^2^ with averages 2, 2, 11

*DWI* diffusion-weighted imaging, *SPAIR* spectral attenuated inversion recovery, *GRAPPA* generalized autocalibrating partially parallel acquisitions

### Data processing

All WB-MRI examinations were evaluated first by staff radiologists as part of their clinical service. Based on clinical information, follow-up imaging, and additional examinations, reported mass lesions were categorized as malignant, benign, or ambiguous, the latter requiring regular follow-up to exclude malignancy. Malignant lesions were confirmed by either histology or follow-up MRI. Ambiguous and malignant lesions were grouped under the category “suspicious.” WB-MRI examinations were categorized as “inconspicuous” (only benign/no lesions) or as “potentially requiring treatment” (at least one suspicious lesion).

Examinations were re-evaluated after a 1-year follow-up to identify lesions that were initially missed but later detected at an increased size. False-positive rates were determined across all MRIs by reviewing further diagnostic workup, including follow-up MRI, but also additional imaging, physical examination or biopsy. False negatives, sensitivity, and specificity were calculated using only examinations with subsequent 1-year follow-up MRI, as this was the sole method to confirm true negatives.

### Visibility rating

In a separate study evaluation, three independent radiologists with 12 years (T.M.), 10 years (F.K.), and 3 years (M.K.) of experience in oncological whole-body MRI scored visibility of mass lesions in each sequence on a Likert scale of 1 to 5 or could choose 0 if the lesion could not be assessed due to severe artifacts (Table 3). Lesions were assessed at the time of their first detection to ensure that malignant lesions were evaluated at their smallest size and that stable benign lesions were not included repeatedly. In case of multiple suspicious lesions of the same organ, only the two biggest lesions per organ were analyzed. The mean visibility score of each sequence was calculated for all mass lesions and for confirmed malignant lesions only. Lesions scored with 4–5 were considered “easily detectable” and with 3 or lower “easily missed” on the respective sequence.

**Table 3:** Likert scale for the evaluation of lesions by readers

	Lesion visibility in each sequence
0	Cannot be evaluated (e.g., due to artifacts)
1	Not detectable
2	Only visible with previous knowledge and/or high contrast windowing
3	Doubtfully visible, can be mistaken for an artifact
4	Visible, low contrast
5	Visible, strong contrast

### Statistical analysis

The sensitivities of each MRI sequence and of all possible 2- and 3-way combinations of sequences were determined by calculating the proportion of lesions classified as “easily detectable.” For lesions located outside of the head, sensitivity calculations were additionally performed for sequence combinations that excluded the FLAIR sequence. A lesion was considered “easily detectable” in a sequence combination if it was categorized as such in at least one of the included sequences. To account for multiple lesions per patient, generalized estimating equations (GEE) logistic regression models, with patients as clusters, were used to estimate sensitivity and specificity along with corresponding 95% confidence intervals (CI). All analyses were done using software R 4.2.1, including add-on packages geepack and emmeans. For the sensitivity calculation of the FLAIR sequence, only lesions located in the head were included, as this was the only region imaged.

## Results

### Study cohort characteristics

Of 116 recruited participants, 113 met the inclusion criteria and underwent WB-MRI evaluations. Two participants were excluded due to claustrophobia and one due to a misclassification of the *TP53* variant (Fig. 1). 91 participants were female (81%) and 22 were male (19%). The age ranged between 19 and 73 years (mean: 40 years; standard deviation: 12.7 years). 59 (52.2%) participants had a history of one malignancy, and 17 (15.0%) of multiple malignancies. Breast cancer was the most common prior diagnosis, reported in 41 participants (36.3%).

In total, 189 WB-MRI examinations, conducted between July 1, 2020, and December 31, 2022, were evaluated. 69 participants (61.1%) were re-examined after 1 year, and 7 participants (6.2%) again after 2 years. Contrast-enhanced sequences were acquired in 186 (98.4%) examinations, with three participants refusing administration of contrast agent.

### Lesion characteristics

20 MRIs with follow-up were classified as “potentially requiring treatment” with at least one suspicious lesion, while 56 exams were categorized as “inconspicuous.”

**Fig. 3:**
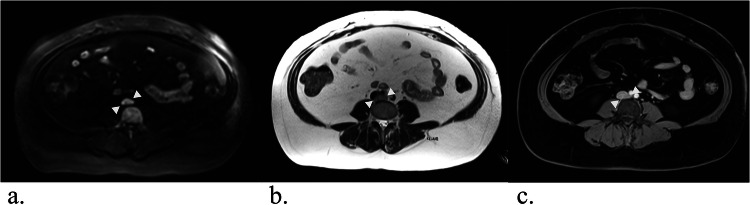
Whole-body MRI of a 34-year-old female study participant with LFS shows a progressive mass behind the inferior vena cava (white arrows). It is very clearly visible in diffusion-weighted imaging (**a**, b-value 900). In the HASTE sequence (**b**), the lesion is somewhat less conspicuous but visible, especially with prior knowledge. The lesion was classified as “easily missed” in the contrast-enhanced T1-weighted Dixon sequence (**c**, here: water-only image), as it was barely distinguishable from the adjacent inferior vena cava. Biopsy revealed a leiomyosarcoma. Images were cropped for better visibility of the lesion

**Fig. 4:**
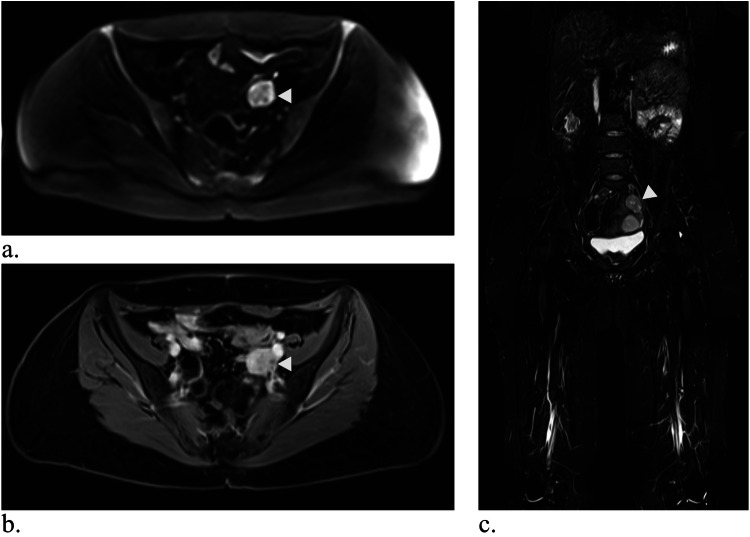
Whole-body MRI of a 39-year-old woman with LFS showing a mass in the small pelvis (white arrow) in diffusion-weighted imaging (**a**) and contrast-enhanced T1-weighted Dixon images (**b**, here: water-only image). In the coronal TIRM-sequence (**c**), the mass is visible, but difficult to differentiate from the intestine, emphasizing the value of diffusion-weighted imaging. Histology revealed a leiomyosarcoma. Images were cropped for better visibility of the lesion

In total, 188 mass lesions were reported in 79 patients (166 at the initial examination, 20 at the 1-year follow-up, 2 at the 2-year follow-up). 120 lesions were classified as benign, 38 as malignant, and 30 as ambiguous. 6 malignant lesions were known prior to the initial WB-MRI, while 32 malignancies (10 primary tumors, 22 metastases) were newly detected in 25 examinations. 140 lesions (*n* = 28 malignant, *n* = 100 benign, *n* = 12 ambiguous) occurred in exams with follow-up and were included in the calculation of sensitivity and specificity. Table 4 provides an overview of lesion characteristics. For a more detailed description of malignant findings, see Supplementary Table 1. Examples of malignant lesions are shown in Figs. 3, 4.

**Table 4:** Lesion characteristics

Final diagnosis	Number of lesions (*N* = 188)
Malignant	38 (20.2%)
Metastasis	26 (13.8%)
Primary tumor	12 (6.4%)
Benign	120 (63.8%)
Uncertain	30 (16.0%)

Lesion size	Size (cm)
Mean (SD)	16.6 (10.9)
Median [IQR]	13.0 [11.23]
Malignant	17.0 [16.25]
Benign	12.0 [9.25]
Uncertain	15.0 [8.75]

*SD* standard deviation, *IQR* interquartile range

### False negatives and sensitivity

**Fig. 5:**
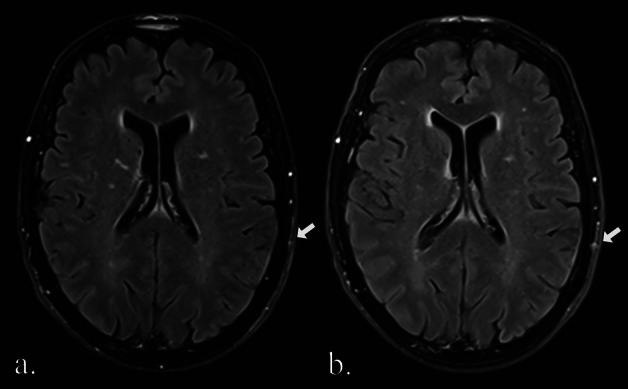
Cutaneous parietal lesion on the left (white arrow), missed in the initial MRI (**a**) and only identified in 1-year follow-up (**b**). In the follow-up examination, the lesion was visible on a single slice exclusively in the FLAIR sequence. Histological analysis following resection confirmed the lesion as a metastasis of the patient’s known metastatic leiomyosarcoma. Images were cropped for better visibility of the lesion

8 lesions were identified in 8 of 76 MRI examinations after 1-year follow-up, that had initially been missed but were retrospectively visible in the original MRI (*n* = 3 malignant, *n* = 2 ambiguous, *n* = 3 benign). Notably, all 5 missed malignant and ambiguous lesions were faint on the initial MRI and may have been retrospectively deemed visible due to prior knowledge during re-evaluation. In 6 cases, at least one other suspicious finding within the same MRI examination prompted further diagnostic workup, leading to the identification and treatment of the missed lesion. The remaining 2 false negatives were ambiguous lesions that were unchanged on follow-up. An example of a missed malignant lesion is presented in Fig. 5.

The false negative rate was 3.5% of MRI examinations with lesions of any dignity and 10% of MRIs with suspicious lesions. Overall, 132 of 140 (94.3%) lesions across all dignities were correctly detected in MRIs with subsequent follow-up, including 35 of 40 (87.5%) suspicious lesions. Of the 20 MRI scans with follow-ups considered as “potentially requiring treatment,” further diagnostic workups were correctly initiated in 18 cases, resulting in a sensitivity rate of 90%.

### False-positives and specificity

43 additional diagnostic tests were recommended in clinical reports of WB-MRIs, including biopsies (*n* = 6), dermatological examinations (*n* = 5), an otolaryngologic examination (*n* = 1), focused additional MRI examinations (*n* = 4 head, *n* = 6 thorax, *n* = 3 whole abdomen, *n* = 7 liver, *n* = 5 lower extremities), vaginal ultrasound (*n* = 3), mammography (*n* = 2), and lung CT (*n* = 1). Among these, 7 diagnostic workups were pending at the time of evaluation. The performed 36 diagnostic tests confirmed 11 lesions requiring treatment (*n* = 10 malignant, *n* = 1 symptomatic benign) and 12 lesions requiring further monitoring. 11 lesions were benign and required no further action, including 4 in MRI exams with follow-up.

**Fig. 6:**
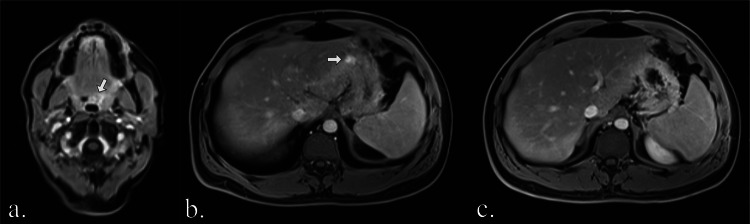
Examples of false positive findings: Contrast-enhancement in the T1 Dixon water-only image of the pharyngeal wall (white arrow in **a**) was confirmed as regular hyperplastic pharyngeal tissue on biopsy. A focal contrast-enhancement of the gastric wall (white arrow in **b**) was no longer detectable on follow-up MRI (**c**) and was retrospectively deemed an artifact. Images were cropped for better visualization

Two reported lesions were deemed false positives as they could not be confirmed: One consisted of a contrast-enhancing thickening of the oropharyngeal wall in a 55-year-old participant, which biopsy revealed to be regular hyperplastic pharyngeal tissue. The other was a focal contrast-enhancement of the gastric wall, which was no longer detectable on follow-up and retrospectively deemed an artifact. Both examples are shown in Fig. 6.

Overall, 94.4% of WB-MRI-triggered additional examinations identified lesions, of which 63.9% required therapy or close monitoring. 1.1% of WB-MRI exams led to unnecessary additional diagnostic investigations. The false positive rate was 3.6% out of 56 inconspicuous exams. Given the increased risk of benign lesions progressing into malignancy in the context of LFS, additional workup of ultimately benign lesions was not classified as false positive. If they are considered as such, the rate of MRIs leading to unnecessary further examinations rises to 6.9% and the false positive rate to 10.7%.

Of 56 MRI exams with follow-up categorized as “inconspicuous,” 50 were accurately reported as such, describing no lesions in 17 cases (17.3%) and only benign lesions in 33 cases (46.7%), resulting in a specificity of 89.3%.

### Reader evaluation

The DWI sequence achieved the best ratings for imaging malignant lesions throughout the entire body (86.0% of lesions categorized as “easily detectable”), while the FLAIR sequence demonstrated the best results for lesions of the head only (100.0% “easily detectable”). 4 malignant lesions that were classified as “easily missed” in DWI by at least one reader were localized in the head and “easily detectable” in the FLAIR sequence (2 low-grade gliomas, 1 brain metastasis, 1 scalp metastasis).

In the evaluation of 2-way combinations, DWI and FLAIR consistently demonstrated the highest ratings for the visualization of malignant lesions. By a 3-way combination of DWI and FLAIR sequence with either the HASTE or the contrast-enhanced T1 sequence, all lesions (100.0%) reported throughout the study period were “easily detectable” in at least one sequence. For lesions located outside of the head, the best 3-way combination was DWI, contrast-enhanced T1, and TIRM. Results for individual sequences and the five best-performing combinations for imaging of malignant lesions are shown in Table 5.

**Table 5:** Sensitivities for malignant lesions, calculated from ratings in the reader evaluation, (a) of individual sequences, (b) of the five best performing combinations of two sequences and (c) of the five best performing combinations of three sequences

Sequence names	Reader 1	Reader 2	Reader 3	Avg
**(a) Individual sequences**				
FLAIR (head only)	100 [NA; NA]	100 [NA; NA]	100 [NA; NA]	100.0
DWI	84.2 [72.7; 91.4]	86.8 [75.7; 93.4]	86.8 [75.7; 93.3]	86.0
TIRM	81.6 [61.3; 92.5]	63.5 [50.6; 74.2]	76.3 [60.3; 87.2]	73.7
T1CE	78.9 [63.7; 88.9]	63.2 [49.0; 79.6]	76.3 [61.2; 86.8]	72.8
HASTE	76.3 [54.2; 89.8]	55.3 [40.3; 69.3]	65.8 [52.7; 76.9]	65.8
T1noCE	60.5 [40.3; 77.7]	55.3 [39.6; 70.0]	73.7 [54.3; 86.8]	63.2
T1cor	57.3 [40.0; 73.9]	47.4 [32.5; 62.7]	52.6 [36.7; 68.0]	52.6
**(b) Combinations of two sequences**				
DWI + FLAIR	94.7 [84.3; 98.4]	94.7 [84.3; 98.4]	97.4 [88.2; 99.5]	95.6
DWI + T1CE	92.1 [82.1; 96.7]	94.7 [81.3; 98.7]	92.1 [82.1; 96.7]	93.0
DWI + HASTE	92.1 [81.5; 96.9]	92.1 [78.5; 97.4]	89.5 [79.4; 94.9]	91.2
DWI + TIRM	89.5 [77.5; 95.4]	89.5 [79.4; 94.9]	92.1 [81.5; 96.9]	90.4
TIRM + T1CE	94.7 [84.3; 98.4]	81.6 [66.3; 90.9]	94.7 [84.3; 98.4]	90.4
**(c) Combinations of three sequences**				
FLAIR + DWI + HASTE	100.0 [NA; NA]	100.0 [NA; NA]	100.0 [NA; NA]	100.0
FLAIR + DWI + T1CE	100.0 [NA; NA]	100.0 [NA; NA]	100.0 [NA; NA]	100.0
FLAIR + DWI+T1cor	97.4 [82.4; 99.7]	97.4 [82.4; 99.7]	100.0 [NA; NA]	98.2
FLAIR + DWI + T1noCE	94.7 [84.3; 98.4]	97.4 [88.2; 99.5]	100.0 [NA; NA]	97.4
FLAIR + DWI + TIRM	94.7 [84.3; 98.4]	97.4 [88.2; 99.5]	97.4 [88.2; 99.5]	96.5
**(d) Combinations of three sequences without FLAIR**				
DWI + T1CE + TIRM	97.4 [81.4; 99.7]	94.7 [80.5; 98.7]	97.4 [81.4; 99.7]	96.5
DWI + T1CE + T1cor	94.7 [80.5; 98.7]	94.7 [80.5; 98.7]	92.1 [81.7; 96.8]	93.9
DWI + HASTE + TIRM	94.7 [79.9; 98.8]	92.1 [77.9; 97.5]	94.7 [79.9; 98.8]	93.9
DWI + HASTE + T1CE	94.7 [83.8; 98.4]	94.7 [80.5; 98.7]	92.1 [81.7; 96.8]	93.9
DWI + T1CE + T1noCE	92.1 [81.7; 96.8]	94.7 [80.5; 98.7]	92.1 [81.7; 96.8]	93.0

Although sensitivities were calculated for all possible sequence combinations, only the five best-performing combinations are displayed here

*FLAIR* fluid-attenuated inversion recovery, *DWI* diffusion-weighted imaging with b-value 900, *TIRM* turbo-inversion recovery-magnitude, *T1CE* contrast-enhanced T1 VIBE Dixon, *HASTE* half Fourier-acquisition single-shot turbo spin echo, *T1noCE* T1 VIBE Dixon without contrast agent, *T1cor* T1 TSE in coronal plane, *NA* not available, *Avg* average

In the analysis of all lesions (benign, ambiguous, and malignant), the FLAIR and DWI sequences, as well as their combination, similarly demonstrated the best results. The best three-sequence combination for all lesions was DWI and the contrast-enhanced T1w sequence with the TIRM sequence. Results for lesions of all dignities can be found in the Supplementary Table 2.

## Discussion

With 113 participants with LFS examined annually with a standardized WB-MRI protocol, this is, to our knowledge, the largest such cohort to date. The entire comprehensive MRI protocol showed an excellent detection of malignant lesions (sensitivity 90%, specificity 89.3%), reaffirming the relevance of WB-MRI in recommendations for LFS surveillance [8].

The reader evaluation of individual MRI sequences demonstrates the potential of an abbreviated WB-MRI protocol for effective cancer detection in individuals with LFS, minimizing examination duration and contrast agent administration. The best results were achieved with a combination of FLAIR (head) with DWI (head to thigh), and either HASTE or a contrast-enhanced T1-weighted DIXON sequence. With both combinations, all malignant lesions identified during the study period were categorized as “easily detectable” in at least one sequence. The best individual sequence was DWI, highlighting its essential role in lesion detection despite its relatively long duration of 24 min.

A significant advantage of an abbreviated protocol is the potential to alleviate patient stress associated with prolonged durations of lying in the MRI scanner. A protocol consisting of the first aforementioned combination (FLAIR, DWI, HASTE) could reduce the duration of the comprehensive protocol by over 50% (41 min) from 77.5 to 36.5 min. The second combination (FLAIR, DWI, contrast-enhanced T1) decreases the scan time to 32.5 min. However, this approach has the drawback of including a contrast agent. While acute side effects are rare [20], there is an ongoing debate about potential long-term gadolinium retention [21]. Although various MRI protocols have been used for LFS surveillance in previous publications, with some taking less than 20 min and including only one or two sequences [7, 22], our findings suggest that such an approach may lack sufficient sensitivity, as no single sequence or combination of two sequences classified all new malignant lesions as “easily detectable.”

Newly detected malignant mass lesions were observed in 5.3% of WB-MRI examinations, which is slightly lower compared to previous studies (e.g., 9% in the UK-SIGNIFY study) [14, 23]. This could be due to differences in the study design, with our study including annual follow-up exams, which tend to detect fewer of previously undiagnosed malignancies compared to baseline examinations, or to differences between MRI protocols.

The false-positive rate in our cohort (3.6%, or 10.7% when considering benign mass lesions as false-positive findings) is substantially lower compared to those reported in other studies. For instance, a meta-analysis by Ballinger et al reported a false-positive rate of 42.5% [14]. This discrepancy may arise from an increased specificity due to our comprehensive MRI protocol, raising suspicion that a shortened protocol could potentially lead to additional follow-up examinations. Another reason may be the high experience of radiologists in our cohort with cancer imaging and surveillance.

This study has several limitations. The relatively small number of malignancies, despite the large cohort size for this rare syndrome, limits the statistical robustness of conclusions, particularly given the diverse tumor spectrum in LFS. Certain malignancies, such as osteosarcomas, were not represented during the study period, despite being part of the core LFS tumor spectrum. This could influence the results of sequence evaluations. While TIRM and T1 TSE sequences, for instance, showed low sensitivity in this study (73.7% and 52.6%, respectively), they are commonly used for bone imaging and may perform better in the detection of bone tumors [24, 25]. Additionally, whole-body MRI protocols for bone assessment often include axial T1 Dixon and DWI sequences. Beyond qualitative assessment, these allow apparent diffusion coefficient (ADC) and fat fraction (FF) mapping as quantitative biomarkers, which may support lesion characterization.

Reader bias is another limitation, as readers were aware of lesion locations prior to visibility scoring and assessed sequences sequentially. While blinded lesion detection was impractical due to the large dataset (> 2000 images per WB-MRI), the use of a Likert scale and evaluations by three independent readers aimed to enhance objectivity. Future studies could assess the diagnostic performance of our best sequence combination through blinded lesion detection, using the full comprehensive protocol as the reference standard.

In the DWI sequence, SPAIR fat suppression was employed due to its higher signal-to-noise ratio and shorter acquisition time compared to STIR, although STIR has been recommended in other studies [15]. Future studies may be warranted to directly compare the performance of both techniques.

The large FOV of 500 mm used here implies prolonged echo trains, which may increase geometrical distortions. While improving geometrical coverage, using a slightly smaller FOV may be justified and could reduce distortions in future studies. Despite the large FOV, upper-extremity assessment remains limited (Supplementary Fig. 1).

In conclusion, an abbreviated MRI protocol including the combination of axial FLAIR (head) with axial HASTE and DWI (head to thigh) offers an effective, contrast-agent-free approach for cancer detection in individuals with LFS. Such a protocol could balance high sensitivity, a reduced duration, and patient safety, making it suitable for long-term annual surveillance. Further studies should aim to assess the utility of the abbreviated protocol with regard to lesion detection, patient stress, and specificity. Despite a high sensitivity, the protocol may be less effective in distinguishing benign from malignant lesions, potentially leading to an increase in additional diagnostic examinations and, therefore, patient burden.

## Supplementary information

ELECTRONIC SUPPLEMENTARY MATERIAL

## Abbreviations

ADC: Apparent diffusion coefficient
ADDRess: Abnormal DNA Damage Response; consortium for the improvement of medical care, diagnostics and therapy for people with impaired DNA repair
DWI: Diffusion-weighted imaging
FLAIR: Fluid-attenuated inversion recovery
FOV: Field of view
HASTE: Half-Fourier acquisition single-shot turbo-spin-echo
LFS: Li-Fraumeni syndrome
MRI: Magnetic resonance imaging
TIRM: Turbo inversion recovery magnitude
TSE: Turbo spin-echo
WB-MRI: Whole-body MRI

## References

[1] Mai PL, Best AF, Peters JA et al (2016) Risks of first and subsequent cancers among TP53 mutation carriers in the National Cancer Institute Li-Fraumeni syndrome cohort. Cancer 122:3673–3681 27496084 10.1002/cncr.30248 PMC5115949

[2] Kamihara J, Rana HQ, Garber JE (2014) Germline TP 53 mutations and the changing landscape of Li-Fraumeni syndrome. Hum Mutat 35:654–662 24706533 10.1002/humu.22559 PMC13577162

[3] Malkin D (2010) Li–Fraumeni syndrome. In: Adrenocortical carcinoma: basic science and clinical concepts. New York: Springer, pp 173–191

[4] Daly MB, Pal T, Maxwell KN, Churpek J, Darlow SD et al (2023). NCCN Guidelines® Insights: Genetic/Familial High-Risk Assessment: Breast, Ovarian, and Pancreatic, Version 2.2024: Featured Updates to the NCCN Guidelines. J Natl Compr Canc Netw 21:1000–1010 10.6004/jnccn.2023.0051 37856201

[5] Ballinger ML, Mitchell G, Thomas DM (2015) Surveillance recommendations for patients with germline TP53 mutations. Curr Opin Oncol 27:332–337 26049273 10.1097/CCO.0000000000000200

[6] McBride KA, Ballinger ML, Killick E et al (2014) Li-Fraumeni syndrome: cancer risk assessment and clinical management. Nat Rev Clin Oncol 11:260–271 24642672 10.1038/nrclinonc.2014.41

[7] Villani A, Shore A, Wasserman JD et al (2016) Biochemical and imaging surveillance in germline TP53 mutation carriers with Li-Fraumeni syndrome: 11 year follow-up of a prospective observational study. Lancet Oncol 17:1295–1305 27501770 10.1016/S1470-2045(16)30249-2

[8] Kratz CP, Achatz MI, Brugières L et al (2017) Cancer screening recommendations for individuals with Li-Fraumeni syndrome. Clin Cancer Res 23:e38–e45 28572266 10.1158/1078-0432.CCR-17-0408

[9] Le AN, Harton J, Desai H et al (2020) Frequency of radiation-induced malignancies post-adjuvant radiotherapy for breast cancer in patients with Li-Fraumeni syndrome. Breast Cancer Res Treat 181:181–188 32246378 10.1007/s10549-020-05612-7 PMC7285877

[10] Anupindi SA, Bedoya MA, Lindell RB et al (2015) Diagnostic performance of whole-body MRI as a tool for cancer screening in children with genetic cancer-predisposing conditions. AJR Am J Roentgenol 205:400–408 26204294 10.2214/AJR.14.13663

[11] Petralia G, Padhani AR (2018) Whole-body magnetic resonance imaging in oncology: uses and indications. Magn Reson Imaging Clin 26:495–507 10.1016/j.mric.2018.06.003 30316463

[12] Daldrup-Link HE, Theruvath AJ, Baratto L, Hawk KE (2022) One-stop local and whole-body staging of children with cancer. Pediatr Radiol 52:391–400 33929564 10.1007/s00247-021-05076-x PMC10874282

[13] Omran M, Johansson H, Lundgren C et al (2023) Whole-body MRI surveillance in TP53 carriers is perceived as beneficial with no increase in cancer worry regardless of previous cancer: data from the Swedish TP53 study. Cancer 129:946–955 36601958 10.1002/cncr.34631

[14] Ballinger ML, Best A, Mai PL et al (2017) Baseline surveillance in Li-Fraumeni syndrome using whole-body magnetic resonance imaging: a meta-analysis. JAMA Oncol 3:1634–1639 28772291 10.1001/jamaoncol.2017.1968 PMC5824277

[15] Petralia G, Koh D-M, Attariwala R et al (2021) Oncologically relevant findings reporting and data system (ONCO-RADS): guidelines for the acquisition, interpretation, and reporting of whole-body MRI for cancer screening. Radiology 299:494–507 33904776 10.1148/radiol.2021201740

[16] Keymling M, Schlemmer H-P, Kratz C et al (2022) Li-Fraumeni syndrome. Radiologie 62:1026–1032 10.1007/s00117-022-01071-x 36166074

[17] Le Bihan D, Breton E, Lallemand D, Aubin ML, Vignaud J, Laval-Jeantet M (1988) Separation of diffusion and perfusion in intravoxel incoherent motion MR imaging. Radiology 168:497–505 3393671 10.1148/radiology.168.2.3393671

[18] Sasamori H, Uno K, Wu J (2019) Usefulness of both PET/CT with F18-FDG and whole-body diffusion-weighted imaging in cancer screening: a preliminary report. Ann Nucl Med 33:78–85 30298377 10.1007/s12149-018-1307-3

[19] Dresen RC, De Vuysere S, De Keyzer F et al (2019) Whole-body diffusion-weighted MRI for operability assessment in patients with colorectal cancer and peritoneal metastases. Cancer Imaging 19:1–10 30616608 10.1186/s40644-018-0187-z PMC6322317

[20] Hunt CH, Hartman RP, Hesley GK (2009) Frequency and severity of adverse effects of iodinated and gadolinium contrast materials: retrospective review of 456,930 doses. AJR Am J Roentgenol 193:1124–1127 19770337 10.2214/AJR.09.2520

[21] Radbruch A, Weberling LD, Kieslich PJ et al (2015) Gadolinium retention in the dentate nucleus and globus pallidus is dependent on the class of contrast agent. Radiology 275:783–791 25848905 10.1148/radiol.2015150337

[22] Gottumukkala RV, Gee MS, Hampilos PJ, Greer M-LC (2019) Current and emerging roles of whole-body MRI in evaluation of pediatric cancer patients. Radiographics 39:516–534 30681900 10.1148/rg.2019180130

[23] Saya S, Killick E, Thomas S et al (2017) Baseline results from the UK SIGNIFY study: a whole-body MRI screening study in TP53 mutation carriers and matched controls. Fam Cancer 16:433–440 28091804 10.1007/s10689-017-9965-1 PMC5487773

[24] Messiou C, Hillengass J, Delorme S et al (2019) Guidelines for acquisition, interpretation, and reporting of whole-body MRI in myeloma: myeloma response assessment and diagnosis system (MY-RADS). Radiology 291:5–13 30806604 10.1148/radiol.2019181949

[25] Smets AM, Deurloo EE, Slager TJE, Stoker J, Bipat S (2018) Whole-body magnetic resonance imaging for detection of skeletal metastases in children and young people with primary solid tumors—systematic review. Pediatr Radiol 48:241–252 29151119 10.1007/s00247-017-4013-8 PMC5790860

[26] Kiermeier S, Schott S, Nees J et al (2024) Health‐related quality of life and fear of progression in individuals with Li‐Fraumeni. J Genet Couns 34:e1859 10.1002/jgc4.1859 PMC11726407 38348940

[27] Nees J, Kiermeier S, Struewe F et al (2022) Health behavior and cancer prevention among adults with Li-Fraumeni syndrome and relatives in Germany—a cohort description. Curr Oncol 29:7768–7778 10.3390/curroncol29100614 PMC9600238 36290891

[28] Penkert J, Strüwe FJ, Dutzmann CM et al (2022) Genotype–phenotype associations within the Li-Fraumeni spectrum: a report from the German Registry. J Hematol Oncol 15:107 10.1186/s13045-022-01332-1 PMC9382737 35974385

