# Supplementary material for: Optimizing whole-body MRI for early cancer detection in Li-Fraumeni syndrome: a prospective bicentric study
Source: Eur Radiol. 2025 Oct 21;36(4):2401–12. doi: 10.1007/s00330-025-11880-y (PMC13035607; doi:10.1007/s00330-025-11880-y)
Supplement: Supplementary file 1 — ELECTRONIC SUPPLEMENTARY MATERIAL [file 330_2025_11880_MOESM1_ESM.docx]

**Supplementary Material**

**Supplementary** **Table 1.** Details of malignant findings.

| Primary tumors (n=11; n=10 would have been undetected without WB-MRI) | | | | | |
| --- | --- | --- | --- | --- | --- |
| Tumor type | **Location** | **Number** | **Histologically confirmed** | **In previous radiation field** | **Symptomatic or known from an examination other than MRI screening** |
| Angiosarcoma | Gluteal muscle | 1 | Yes | Yes | Yes |
| Adenoid cystic carcinoma | Lacrimal gland | 1 | Yes | No | No |
| Breast cancer | Right mamma | 2 | Yes | No | No |
| Leiomyosarcoma | - Retroperitoneal (n=2) - Small pelvis (n=1) | 3 | Yes | No | No |
| Low-grade glioma | Frontal lobe (n=2) | 2 | No | No | No |
| Pancreatic cancer | Pancreatic tail | 1 | Yes | No | No |
| Pleomorphic sarcoma | Quadriceps muscle | 1 | Yes | Yes | No |
| Metastases (n=27; n=22 would have been undetected without WB-MRI) | | | | |  |
| Organ | **Exact location** | **Number** | **Histologically confirmed** | **In previous radiation field** | **Symptomatic or known from an examination other than MRI screening** |
| Bone | - 7^th^ thoracic vertebra (n=1) - 9^th^ thoracic vertebra (n=1) - 1^st^ lumbar vertebra (n=1) - Os ilium (n=1) | 4 | No | No | No |
| Lymph nodes | - Axillary (n=1) - Subpectoral (n=2) - Retroperitoneal (n=3) - Small pelvis (n=3) | 9 | Subpectoral: yes (n=1)  Others: No | No | Axillary: yes (n=1)  Others: no |
| Lung | - Lower lobe of left lung (n=1) - Lower lobe of right lung (n=1) | 2 | No | No | Yes |
| Liver | - Segment VII (n=2) - Segment II (n=1) | 3 | No | No | Segment VII: Yes (n=1)  Others: No (n=2) |
| Brain | - Temporal lobe (n=2) - Cerebellum (n=1) | 3 | No | No | No |
| Fatty tissue | Paracolic gutter | 1 | No | No | Yes |
| Skin | - Frontal scalp (n=2) - Gluteal (n=2) | 4 | No | No | No |
| Muscle | - 11^th^ intercostal space left | 1 | No | No | No |

**Supplementary** **Table *2*.** Sensitivities for all lesions including benign lesions, calculated from ratings in the reader evaluation, a) of individual sequences, b) of the five best performing combinations of two sequences and c) of the five best performing combinations of three sequences.

| Sequence names | Reader 1 | Reader 2 | Reader 3 | Avg |
| --- | --- | --- | --- | --- |
| 1. Individual sequences | | | | |
| FLAIR (head only) | 92.9 [62.5;99.0] | 94.4 [73.7;99.0] | 100.0 [100.0;100.0] | 95.8 |
| DWI | 86.7 [81.2;90.8] | 70.2 [63.1;76.5] | 77.5 [71.0;83.0] | 78.2 |
| TIRM | 73.9 [65.3;81.1] | 63.8 [56.9;70.2] | 71.8 [64.2;78.4] | 69.9 |
| T1CE | 79.3 [71.8;85.2] | 61.7 [53.5;69.3] | 67.0 [57.4;75.4] | 69.3 |
| HASTE | 76.6 [68.7;83.0] | 59.0 [52.4;65.3] | 69.0 [62.5;74.8] | 68.2 |
| T1noCE | 60.6 [52.6;68.1] | 46.8 [39.6;54.2] | 62.2 [54.8;69.1] | 56.6 |
| T1cor | 50.5 [41.9;59.2] | 34.6 [27.9;41.9] | 46.3 [39.0;53.8] | 43.8 |
| 1. Combinations of two sequences | | | | |
| DWI.T1CE | 96.3 [92.8;98.1] | 86.7 [81.0;90.9] | 91.4 [86.8;94.6] | 91.5 |
| DWI.TIRM | 92.6 [87.3;95.7] | 89.9 [85.1;93.3] | 90.9 [85.8;94.3] | 91.1 |
| DWI.HASTE | 93.6 [89.3;96.3] | 86.2 [80.7;90.3] | 88.8 [83.7;92.4] | 89.5 |
| T1CE.HASTE | 92.0 [86.5;95.4] | 80.9 [74.5;85.9] | 88.2 [83.0;92.0] | 87.0 |
| T1CE.TIRM | 89.4 [83.2;93.5] | 80.9 [74.2;86.1] | 89.4 [82.6;93.7] | 86.5 |
| 1. Combinations of three sequences | | | | |
|  | Reader 1 | Reader 2 | Reader 3 | Avg |
| DWI.T1CE.TIRM | 97.3 [93.8;98.9] | 94.1 [90.0;96.6] | 96.3 [91.6;98.4] | 95.9 |
| DWI.T1CE.HASTE | 97.9 [94.7;99.2] | 94.1 [89.9;96.7] | 94.7 [90.8;96.9] | 95.6 |
| DWI.T1CE.FLAIR | 99.5 [96.4;99.9] | 89.9 [84.2;93.7] | 94.7 [90.0;97.2] | 94.7 |
| DWI.TIRM.HASTE | 95.2 [90.8;97.6] | 94.7 [90.3;97.1] | 94.1 [89.3;96.8] | 94.7 |
| DWI.TIRM.FLAIR | 95.2 [91.0;97.5] | 93.6 [89.2;96.3] | 93.6 [88.8;96.4] | 94.1 |
| FLAIR = fluid-attenuated inversion recovery, DWI = diffusion-weighted imaging with b-value 900, TIRM = turbo-inversion recovery-magnitude, T1CE = contrast-enhanced T1 VIBE Dixon, HASTE = half Fourier-acquisition single-shot turbo spin echo, T1noCE = T1 VIBE Dixon without contrast agent, T1cor = T1 TSE in coronal plane | | | | |


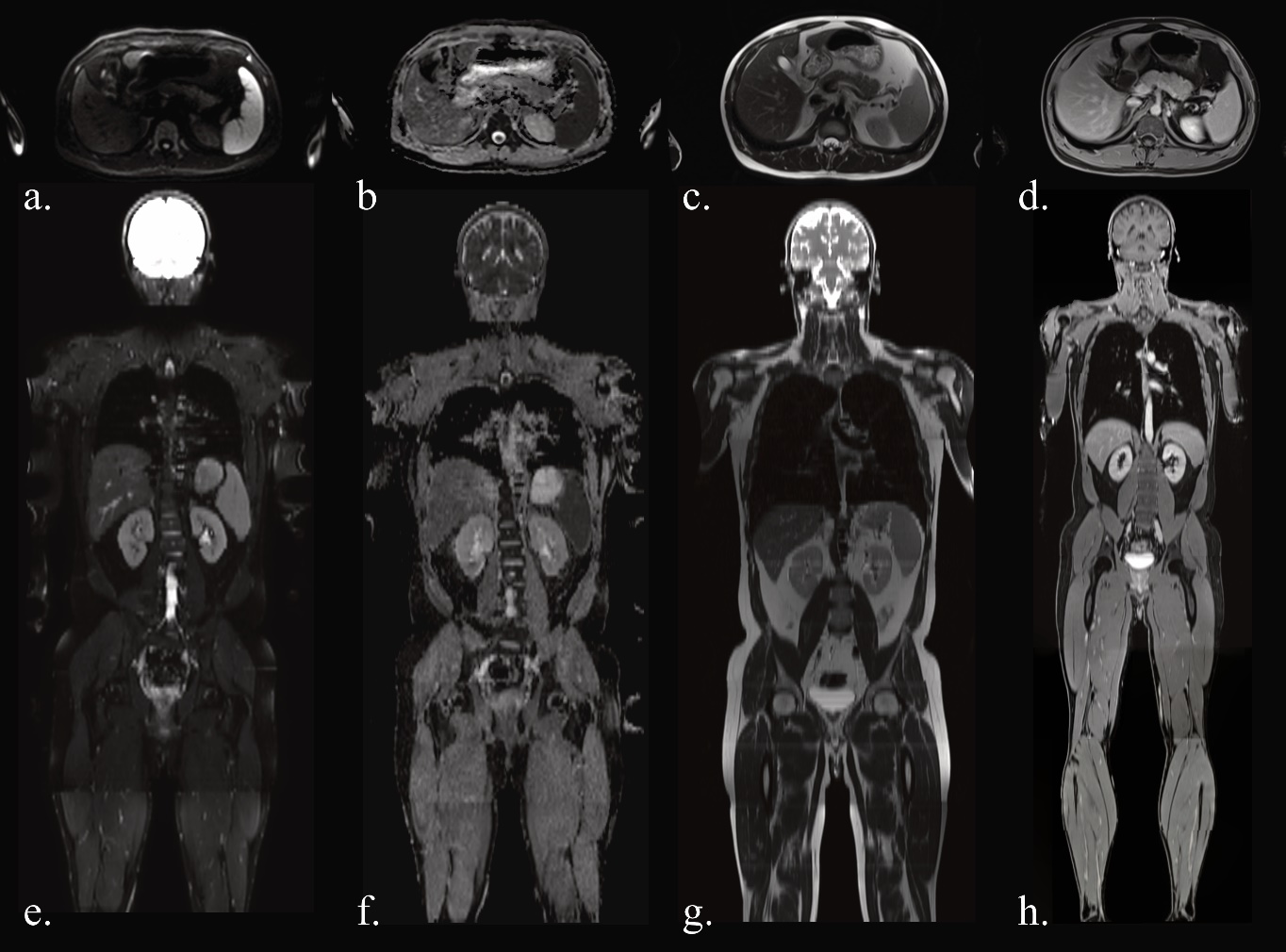


**Supplementary Figure 1.** Examples of uncropped images: DWI at b-value 900 (axial reconstruction: a, coronal reconstruction: e), ADC map (axial: b, coronal: f), HASTE (axial: c, coronal: g) and T1 VIBE Dixon water-only image after contrast agent administration (axial: d, coronal: h). Although the assessment of the upper extremities is limited due to partial coverage and artifacts, the clinical impact of this is likely limited.
